# Impact of persistence on survival of patients with breast cancer treated with endocrine therapy in Northeast China: a prospective study

**DOI:** 10.18632/oncotarget.18454

**Published:** 2017-06-12

**Authors:** Peng Xing, Huiting Dong, Qun Liu, Fan Yao, Yingying Xu, Bo Chen, Xinyu Zheng, Yunfei Wu, Feng Jin, Jiguang Li

**Affiliations:** ^1^ Breast Division, The First Hospital of China Medical University, Shenyang 110001, Liaoning, People's Republic of China

**Keywords:** breast cancer, endocrine therapy, treatment persistence, risk factors

## Abstract

The purpose of this prospective study is to investigate the impact of endocrine treatment persistence on the survival of patients with estrogen receptor-positive breast cancer treated with endocrine therapy and identify the risk factors influencing the treatment persistence. We enrolled 1085 patients from Northeast China who were diagnosed as stage I–III, estrogen receptor-positive breast cancer between January 2007 and December 2010. The prognostic factors for disease-free survival (DFS) and overall survival (OS) of patients were identified using univariate and multivariate Cox proportional hazards regression models. Multiple logistic regression analysis was done to determine the possible risk factors for non-endocrine treatment and treatment discontinuation. Among the patients enrolled, 598 (55.1%) underwent 5 years of endocrine therapy, 278 (25.6%) less than 5 years, and 209 (19.3%) non-endocrine therapy. OS rates in the continuation, discontinuation, and non-endocrine treatment groups were 97.8%, 92.6% and 74.3%, and DFS 97.5%, 86.2% and 69.9%, respectively. After adjusting for pathological and socioeconomic factors, non-endocrine therapy and discontinuation were independent predictors for DFS and OS. Elderly patients (≥ 65 years), those living in suburban and rural areas, locally advanced patients, and receiving no radiotherapy and/or chemotherapy were more likely to receive non-endocrine therapy and discontinue endocrine treatment. In conclusion, the prospective study demonstrate that the persistence of endocrine treatment is low in estrogen receptor-positive breast cancer patients in Northeast China. Non-endocrine treatment and early discontinuation serve as independent prognostic factors for both DFS and OS of breast cancer patients treated with endocrine therapy.

## INTRODUCTION

Breast cancer is the most common malignancy in women worldwide and the most frequent cause of cancer-related death in females [1]. The incidence and mortality rates of breast cancer have been increasing rapidly since 1980s [2]. Endocrine therapy is an efficacious treatment option for estrogen receptor positive (ER+) breast cancer, which can significantly reduce the recurrence rate and mortality [3]. However, long-term of endocrine therapy is the key to ensure the efficacy. Previous study have found that 3 years or even 5 years of treatment with tamoxifen is significantly more effective than shorter tamoxifen regimens (1 year) [4, 5]. Recently, newer statistics from two large studies, the Adjuvant Tamoxifen: Longer Against Shorter (ATLAS) trial and the Adjuvant Tamoxifen—To Offer More? (aTTom), have confirmed that longer-term tamoxifen use (for up to 10 years) rather than stopping at 5 years generates a further reduction in recurrence and mortality [6, 7].

Poor medication compliance and persistence is a major issue affecting all chronic diseases [8]. Breast cancer is no exception. Compliance is commonly defined as taking medication as directed (e.g., at a certain time of the day), whereas persistence is generally defined as continuing to take medication (correctly or incorrectly) for the recommended period. Adherence refers to the overall behavior, which includes persistence and compliance [9, 10]. Although the benefits of endocrine therapy on ER+ breast cancer, the adherence and persistence are rather poor [11, 12]. Van-Herk-Sukel *et al.* reported that about 50% of the breast cancer patients discontinued tamoxifen or any endocrine treatment before the recommended treatment period of 5 years [13]. Hershman *et al.* suggested that only 49% of breast cancer patients took adjuvant endocrine therapy for the full duration [14]. Early termination of endocrine therapy might increase the risk of breast cancer associated mortality [12, 15, 16]. Discontinuation of endocrine therapy is mainly due to demographic/medical variables, modifiable psychosocial characteristics, and the side effects [17–19]. However, the evidence is mainly from Western countries. The peak ages of breast cancer patients in the Western countries are mainly at 60–70 years old, which is 10 years later than the patients in the Asian countries [20, 21].

Therefore, in the present study, we aimed to investigate the persistence patterns for women patients with ER+ breast cancer receiving hormonal therapy in Northeast China. The patients were grouped according to the treatment patterns: non-endocrine treatment, discontinuation, and continuation. The effects of different patterns on patients’ survival were explored, and then the underlying influence factors and reasons for non-endocrine treatment and discontinuation were tried to discover.

## RESULTS

### Response rate

From January 2007 to December 2010, a total of 1431 women were diagnosed with hormone receptor-positive stage I–III breast cancer at our department. We excluded patients with neoadjuvant chemotherapy (92 cases), carcinoma *in situ* (332 cases), disease progression during endocrine therapy (74 cases), and other malignant diseases (6 cases). A total of 1085 patients were enrolled in the present study. Over the 5 year follow-up period, 986 patients were successfully followed and 99 patients were lost to follow-up. The response rate was 90.8%. There were respectively 7, 6, 10, 14, 13, 12, 10, 11, and 16 patients were lost from the first follow-up to the last one.

### Demographic and clinical characteristics of the patients

Baseline demographic and clinical characteristics of the patients were shown in Table 1. Patients were categorized into three groups: non-endocrine treatment, early discontinuation, and continuation. The median age of the patients was 51 years (20–85 years). In total, 876 patients received endocrine therapy, i.e., 631 tamoxifen only, 183 AI only, and 62 both. Early discontinuation of endocrine therapy was noted in 278 cases (31.7%). No statistical differences were detected with respect to the family history of breast/ovarian cancer (*P* = 0.670), history of gynecologic benign diseases (*P* = 0.210), radiotherapy status (*P* = 0.060), and median follow-up (*P* = 0.808). We observed that there were significant differences in ages (*P* = 0.000), residence (*P* = 0.000), surgery type (*P* = 0.000), tumor grade (*P* = 0.000), lymph node involvement (*P* = 0.000), and chemotherapy (*P* = 0.000) among the three groups. At the end of follow-up, 96 patients died from breast cancer and 21 patients died from non-cancer causes. Additionally, 54 patients were alive at the end of the study, but developed recurrence of the disease.

**Table 1:** Baseline characteristics of patients with hormone-receptor-positive breast cancer

Characteristics, No. of patients (%)	Non-endocrine therapy	Endocrine therapy		*P* value
		Discontinuation	Continuation	
Ages at diagnosis, years				0.000
< 50	69 (33.0)	144 (51.8)	281 (47.0)	
50–64	84 (40.2)	106 (38.1)	256 (42.8)	
≥ 65	56 (26.8)	28 (10.1)	61 (10.2)	
Residence				0.000
Urban	89 (42.6)	156 (56.1)	442 (73.9)	
Suburban and rural	120 (57.4)	122 (43.9)	156(26.1)	
Surgery				0.000
Tumorectomy	21 (10.0)	12 (4.3)	26 (4.3)	
Breast-conserving	7 (3.4)	21 (7.6)	51 (8.5)	
Modified radical mastectomy	181 (86.6)	245 (88.1)	521 (87.2)	
Family history of breast/ovarian cancer				0.670
Yes	7 (3.4)	14 (5.0)	24 (4.0)	
No	202 (96.6)	264 (95.0)	574 (96.0)	
History of gynecologic benign diseases				0.210
Yes	31 (14.8)	39 (14.0)	110 (18.4)	
No	178 (85.2)	239(86.0)	488 (81.6)	
Tumor grade				0.000
T1	66 (31.6)	122 (43.9)	288 (48.2)	
T2	128 (61.2)	139 (50.0)	288 (48.2)	
T3	14 (6.7)	15 (5.4)	18 (3.0)	
Unknown	1 (0.5)	2 (0.7)	4 (0.6)	
Lymph node				0.000
N0	78 (37.3)	150 (54.0)	341 (57.0)	
N1	43 (20.6)	66 (23.7)	147 (24.6)	
N2	37 (17.7)	26 (9.4)	59 (9.9)	
N3	30 (14.4)	21 (7.6)	23 (3.8)	
Unknown	21 (10.0)	15 (5.3)	28 (4.7)	
Chemotherapy				0.000
Yes	104 (49.8)	228 (82.0)	465 (77.8)	
No	105 (50.2)	50 (18.0)	133 (22.2)	
Radiotherapy				0.060
Yes	38	68	158	
No	171	210	440	
Median follow-up (months)^*^	88	84	86	0.808

TNM, tumor node metastasis.

^*^Differences were analyzed using the Kruskal-Wallis test.

### Survival analysis

**Figure 1:**
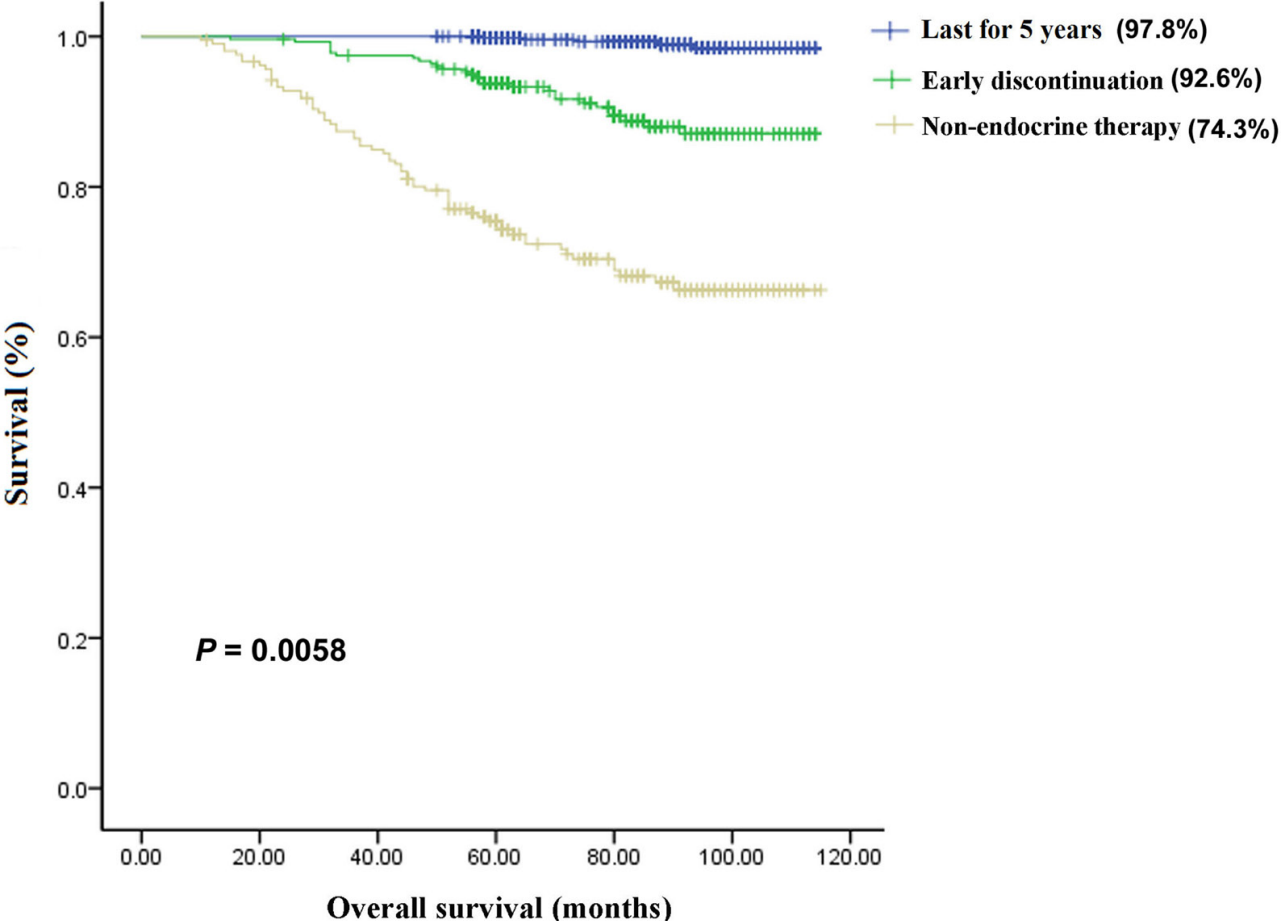
The 5-year overall survival (OS) rates in the three groups The 5-year OS rates were 74.3%, 92.6%, and 97.8%, respectively, in the non-endocrine treatment (*n* = 209) group, early discontinuation (*n* = 278) group, and continuation (*n* = 598) group, and there were significant differences among the three groups.

**Figure 2:**
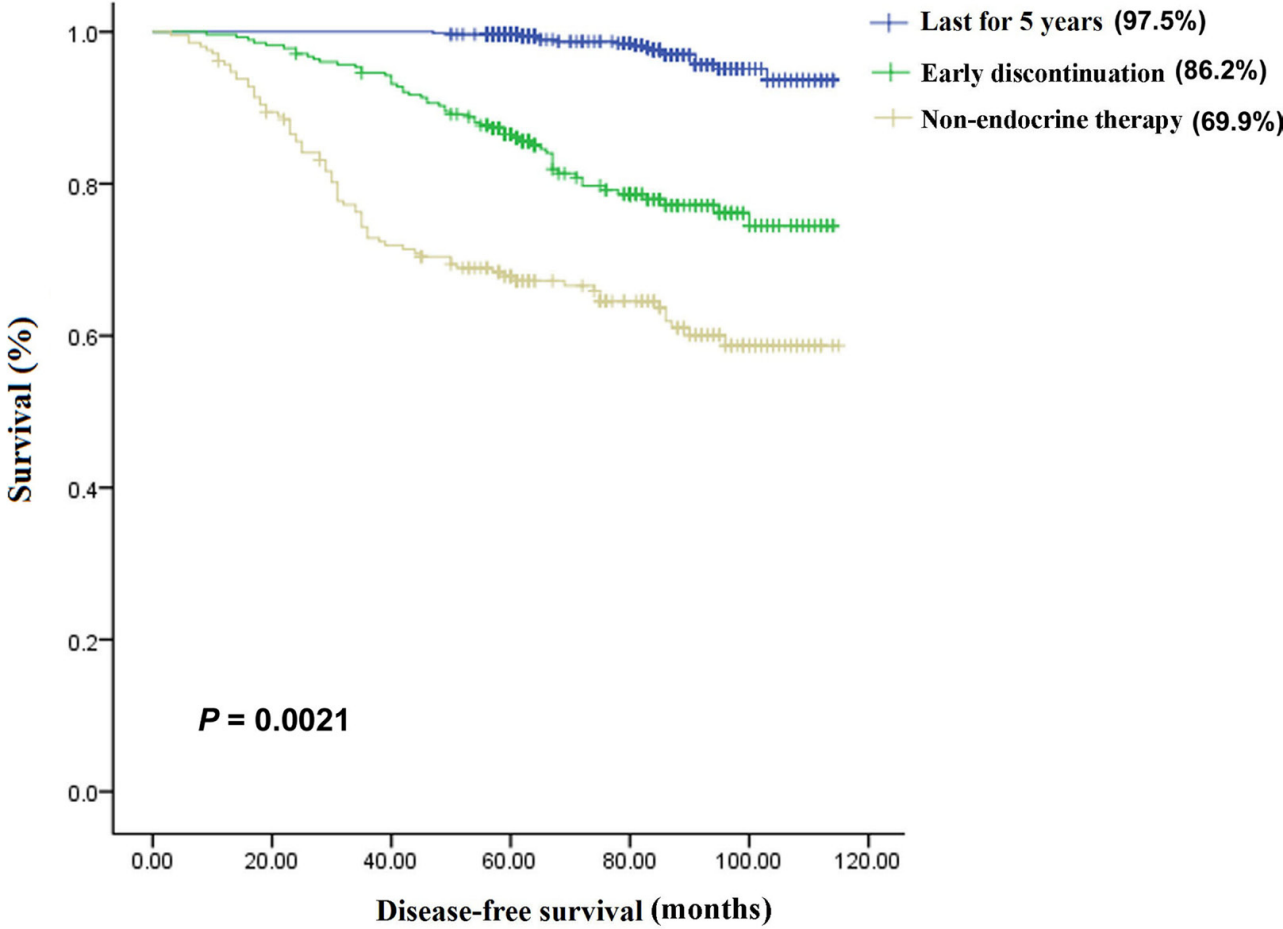
The 5-year disease-free survival (DFS) rates in the three groups The 5-year DFS rates were 69.9%, 86.2%, and 97.5%, respectively, in the non-endocrine treatment (*n* = 209) group, early discontinuation (*n* = 278) group, and continuation (*n* = 598) group, and there were significant differences among the three groups.

The 5-year overall survival (OS) rates were 74.3%, 92.6%, and 97.8% in the non-endocrine treatment, discontinuation, and continuation groups, respectively (*P* = 0.0058; Figure 1), whereas the 5-year disease-free survival (DFS) rates were 69.9%, 86.2%, and 97.5%, respectively (*P* = 0.0021; Figure 2). The predictors of OS and DFS were further analyzed by univariate and multivariate Cox proportional hazards regression (Table 2). After adjustment for clinical pathological factors and social economic factors, we confirmed that non-endocrine treatment and discontinuation still remained independent predictors for DFS (non-endocrine treatment: hazard ratio (HR), 13.180; 95% confidence interval (CI), 7.610-22.824; *P* = 0.000; discontinuation: HR, 7.621; 95% CI, 4.410–13.167; *P* = 0.000) and OS (non-endocrine treatment: HR, 28.080; 95% CI, 11.017–71.571; *P* = 0.000; discontinuation: HR, 10.976; 95% CI, 4.215–28.582; *P* = 0.000).

**Table 2:** Univariate and multivariate analysis of DFS and OS

Characteristics	DFS				OS			
	Univariate HR (95% CI)	*P* value	Multivariate HR (95% CI)	*P* value	Univariate HR (95% CI)	*P* value	Multivariate HR (95% CI)	*P* value
Ages at diagnosis, years								
< 50	1.000 -		1.000 -	-	1.000 -	-	1.000 -	-
50–64	1.105 (0.779–1.567)	0.575	1.031 (0.720–1.478)	0.866	1.073 (0.692–1.663)	0.754	0.846 (0.536–1.337)	0.475
≥ 65	1.372 (0.857–2.198)	0.188	0.823 (0.477–1.420)	0.484	1.404 (0.786–2.507)	0.251	0.621 (0.319–1.210)	0.161
Radiotherapy								
No	1.000 -	-	1.000 -	-	1.000 -	-	1.000 -	-
Yes	1.73 (1.235–2.421)	0.001	0.891 (0.574–1.382)	0.606	1.602 (1.048–2.449)	0.03	0.877 (0.517–1.487)	0.625
Her-2 status								
Negative	1.000 -		1.000 -	-	1.000 -	-	1.000 -	-
Positive	1.487 (1.026–2.155)	0.036	1.420 (0.965–2.090)	0.075	1.342 (0.834–2.161)	0.23	1.302 (0.792–2.141)	0.299
Surgery								
Tumorectomy	1.000 -	-	1.000 -	-	1.000 -	-	1.000 -	-
Breast-conserving	0.532 (0.198–1.429)	0.211	0.161 (0.035–0.752)	0.020	0.348 (0.087–1.390)	0.135	0.11 (0.017–0.700)	0.019
MRD/Total mastectomy+SLN	0.847 (0.432–1.665)	0.631	0.114 (0.029–0.448)	0.002	0.838 (0.366–1.916)	0.675	0.099 (0.022–0.446)	0.003
T stage								
T1	1.000 -	-	1.000 -	-	1.000 -	-	1.000 -	-
T2	3.272 (2.181–4.907)	0.000	1.953 (1.259–3.028)	0.003	5.985 (3.175–11.281)	0.000	2.946 (1.488–5.833)	0.002
T3	5.075 (2.647–9.732)	0.000	2.237 (1.119–4.471)	0.023	11.08 (4.803–25.560)	0.000	3.545 (1.463–8.592)	0.005
Unknown	2.542 (0.347–18.644)	0.359	3.016 (0.404–22.522)	0.282	6.81 (0.879–52.765)	0.066	3.786 (0.457–31.371)	0.217
N stage								
N0	1.000 -	-	1.000 -	-	1.000 -	-	1.000 -	-
N1	1.598 (0.988–2.584)	0.056	1.470 (0.904–2.390)	0.120	1.953 (0.984–3.876)	0.056	1.735 (0.870–3.461)	0.118
N2	4.57 (2.900–7.203)	0.000	3.732 (2.196–6.344)	0.000	7.064 (3.833–13.019)	0.000	4.938 (2.493–9.781)	0.000
N3	12.046 (7.775–18.663)	0.000	7.386 (4.384–12.444)	0.000	22.027 (12.447–38.980)	0.000	9.605 (5.052–18.259)	0.000
Unknown	1.713 (0.768–3.819)	0.188	0.195 (0.039–0.985)	0.048	2.204 (0.746–6.514)	0.153	0.166 (0.025–1.117)	0.065
Chemotherapy								
No	1.000 -	-	1.000 -	-	1.000 -	-	1.000 -	-
Yes	0.714 (0.506–1.006)	0.054	1.033 (0.677–1.578)	0.880	0.512 (0.340–0.771)	0.001	0.758 (0.458–1.253)	0.280
Endocrine therapy								
Non-endocrine treatment	16.864 (9.970–28.526)	0.000	13.180 (7.610–22.824)	0.000	44.144 (17.742–109.686)	0.000	28.080 (11.017–71.571)	0.000
Discontinuation	7.824 (4.546–13.465)	0.000	7.621 (4.410–13.167)	0.000	12.696 (4.902–32.880)	0.000	10.976 (4.215–28.582)	0.000
Continuation	1.000-	-	1.000-	-	1.000-	-	1.000-	-

HR, hazard rate; CI, confidence interval; AI, aromatase inhibitors; MRD, modified radical mastectomy; SLN, sentinel lymph node biopsy.

### Risk factors for non-endocrine treatment and discontinuation

We further performed logistic regression analysis to identify the possible risk factors of non-endocrine treatment and discontinuation (Table 3). As described in the table, we observed that compared with patients who were less than 50 years old, patients more than 65 years old were one risk factor for non-endocrine therapy (odds ratio (OR), 4.204; 95% CI, 2.268–7.792; *P* = 0.000) and discontinuation (OR, 2.179; 95% CI, 1.074–4.420; *P* = 0.031). Compared to patients who lived in urban area, patients who lived in suburban and rural area were risk factors for non-endocrine therapy (OR, 4.278; 95% CI, 2.880–6.355; *P* = 0.000) and discontinuation (OR, 1.948; 95% CI, 1.412–2.687; *P* = 0.000). Compared with patients with T1 stage, T3 stage was a risk factor for discontinuation (OR, 2.331; 95% CI, 1.061–5.122; *P* = 0.035). Compared with patients with N0 stage, N2 stage and N3 stage were risk factors for non-endocrine therapy (N2 stage: OR, 6.614; 95% CI, 3.200–13.671; *P* = 0.000; N3 stage: OR, 11.279; 95% CI, 4.826–26.362; *P* = 0.000), and while only N3 stage was a risk factor for discontinuation (OR, 1.379; 95% CI, 1.239–5.963; *P* = 0.013). Moreover, the results showed that patients who did not received radiotherapy and/or chemotherapy were risk factors for non-endocrine therapy (no radiotherapy: OR, 4.723; 95% CI, 2.479–8.998; *P* = 0.000; no chemotherapy: OR, 2.065; 95% CI, 1.350–3.160; *P* = 0.001) and discontinuation (no radiotherapy: OR, 1.753; 95% CI, 1.032–2.977; *P* = 0.038; no chemotherapy: OR, 1.602; 95% CI, 1.048–2.448; *P* = 0.030). Interestingly, we observed that the types of endocrine therapy were also related with discontinuation. Tamoxifen only was a risk factor for discontinuation (OR, 2.351; 95% CI, 1.450–3.812; *P* = 0.001). However, type of surgery, family history of breast/ovarian cancer, and history of gynecologic benign diseases had no significant association with the non-endocrine therapy and discontinuation.

**Table 3:** Logistic regression analysis of risk factors associated with non-endocrine treatment and early discontinuation

Characteristics	Non-endocrine treatment		Discontinuation	
	OR (95%CI)	*P* value	OR (95%CI)	*P* value
Ages at diagnosis, years				
< 50	1.000-	-	1.000-	-
50–64	1.408 (0.926–2.142)	0.109	1.14 (0.813–1.599)	0.448
≥ 65	4.204 (2.268–7.792)	0.000	2.179 (1.074–4.420)	0.031
Residence				
Urban	-		1.000-	-
Suburban and rural	4.278 (2.880–6.355)	0.000	1.948 (1.412–2.687)	0.000
Surgery				
Tumorectomy	1.064 (0.110–10.318)	0.957	0.612 (0.127–2.943)	0.540
Breast-conserving	1.505 (0.575–3.934)	0.405	1.286 (0.674–2.453)	0.445
Modified radical mastectomy	-	-	1.000-	-
Family history of breast/ovarian cancer				
Yes	1.800 (0.659–4.911)	0.251	1.526 (0.756–3.081)	0.238
No	-	-	1.000-	-
History of gynecologic benign diseases				
Yes	1.307 (0.785–2.175)	0.304	0.861 (0.568–1.305)	0.481
No	-		1.000-	-
T stage				
T1	-		1.000-	-
T2	1.400 (0.940–2.085)	0.098	1.065 (0.777–1.459)	0.695
T3	1.963 (0.706–5.462)	0.196	2.331 (1.061–5.122)	0.035
Unknown	0.675 (0.053–8.625)	0.763	0.857 (0.146–5.045)	0.865
N stage				
N0	-	-	1.000-	-
N1	1.288 (0.804–2.064)	0.293	0.991 (0.684–1.436)	0.961
N2	6.614 (3.200–13.671)	0.000	1.379 (0.709–2.682)	0.344
N3	11.279 (4.826–26.362)	0.000	1.379 (1.239–5.963)	0.013
Unknown	2.123 (0.208–21.637)	0.525	1.379 (0.652–11.806)	0.167
Radiotherapy				
Yes	-	-	1.000-	-
No	4.723 (2.479–8.998)	0.000	1.753 (1.032–2.977)	0.038
Chemotherapy				
Yes	-	-	1.000-	-
No	2.065 (1.350–3.160)	0.001	1.602 (1.048–2.448)	0.030
Types of endocrine therapy				
Tamoxifen only	-	-	2.351 (1.450–3.812)	0.001
AI only	-	-	1.000-	-
Tamoxifen+ AI	-	-	0.970.419–2.249)	0.944

OR, odds ratio; CI, confidence interval; AI, aromatase inhibitors.

### Reasons for patients not receiving endocrine therapy

Among the 1085 patients, there were 209 patients who had not received endocrine therapy. The common reasons were listed as follows (Table 4): (a) no confidence in endocrine therapy (*n* = 132); (b) advanced age or accompanied by other cardiovascular and cerebrovascular disease (*n* = 50); (c) fearing of side effects caused by endocrine therapy (*n* = 12); (d) switching to traditional Chinese medicine (*n* = 8); and (e) economic or other causes (*n* = 7).

**Table 4:** Reasons for patients not receiving endocrine therapy

Reasons	No. (%)
Patients had no confidence in endocrine therapy	132 (63.16)
Patients with advanced age or accompanied by other cardiovascular and cerebrovascular disease	50 (23.92)
Patients were fear of side effects caused by endocrine therapy	12 (5.74)
Patients did not trust the endocrine therapy but turned to Traditional Chinese Medicine	8 (3.83)
Patients could not afford or were not easily obtain the drugs due to the financial or residential conditions	7 (3.35)

### Reasons for early discontinuation of endocrine therapy

There were a total of 278 patients who early discontinued the endocrine therapy. The percentages of patients who continued endocrine therapy were respectively 93.2%, 86.2%, 78.8%, 72.3%, and 68.3% from the first year to the fifth year. The reasons were listed as below (Table 5): (a) presence of side effects (*n* = 134) including endometrial thickening (*n* = 48), facial flush, sweat, and weakness (*n* = 23), and gastrointestinal discomfort and limb ache (*n* = 63); (b) feeling no need to continue treatment due to good outcome (*n* = 122); (c) patients with advanced ages or accompanied by other cardiovascular and cerebrovascular disease (*n* = 12); (d) switching to traditional Chinese medicine (*n* = 5); and (e) economic or other causes (*n* = 5).

**Table 5:** Reasons for patient's early discontinuing endocrine therapy

Reasons	No. (%)
Patients presented side effects	134 (48.20)
Endometrial thickening	48 (17.27)
Facial flush, Sweat, and Weakness	23 (8.27)
Gastrointestinal discomfort and Limb ache	63 (22.66)
Patients felt well and thought no need to continue the endocrine therapy	122 (43.88)
Patients with advanced ages or accompanied by other cardiovascular and cerebrovascular disease	12 (4.32)
Patients turned to Traditional Chinese Medicine	5 (1.80)
Patients could not afford or were not easily obtain the drugs due to the financial or residential conditions	5 (1.80)

## DISCUSSION

Endocrine therapy has been identified as an effective treatment for ER+ breast cancer, with a recommended standard time for at least 5 years. Nevertheless, the compliance and persistence to medications is rather poor. In the prospective study, we confirmed that the persistence of endocrine treatment was also poor in women patients with ER+ breast cancer in Northeast China. Non-endocrine treatment and early discontinuation were independent predictors for both DFS and OS. Elderly women, patients who lived in suburban and rural areas, locally advanced patients, and patients who received no radiotherapy and/or chemotherapy were significantly detected in patients with non-endocrine treatment and early discontinuation.

It has been well documented that surgery followed by adjuvant treatment is a gold standard for breast cancer treatment [22]. Approximately 80% of breast cancers are ER+ and thus endocrine therapy is regarded as an important complement to surgery in the majority of patients [23]. Currently endocrine therapy includes gonadotropin-releasing hormone agonists (GnRHa), selective estrogen receptor modulators (SERMs) or down-regulators (SERDs), and aromatase inhibitors (AIs), or a combination [23]. Although the benefits of receiving endocrine therapy, a growing body of evidence suggest that discontinuation of endocrine therapy has a negative impact on recurrence and mortality of breast cancer [14, 15, 24]. For example, a recent study has shown that the earlier termination of chemotherapy, the higher risk of breast cancer related death [14]. However, the available evidence suggested that only 30% to 50% of women were fully complete with hormonal therapy for 5 years [25–32]. In our study, the discontinuation rate was 55.1%, which was a little higher than the previous above studies. A possible explanation for this phenomenon was the response and/or self-presentation bias. Some reliable data might not be able to directly access from patents because of these biases. In spite of this, improving of the follow-up quality may reduce the biases in our study. Although the relatively persistence rate at the beginning of the endocrine therapy in our study, the rate decreased year by year from 93.2% in the first year to 68.2% in the fifth year. Our results were in line with previous studies [25, 27, 33] showing that about 7–10% patients discontinued hormonal therapy (either tamoxifen or AI) every year. In addition, our data also revealed that non-endocrine therapy and early discontinuation were independent r predictors of breast cancer related death, which were consistent with previous studies [12, 15, 34].

We further analyzed the potential risk factors of non-endocrine treatment and discontinuation by univariate and multivariate Cox proportional hazards regression models. Among all the risk factors, the ages of patients appeared to be very important. No significant difference was found in the persistence of among younger patients (< 50 years) and patients who were 50–65 years old. But interestingly, we observed that the elderly patients were one of the risk factors of non-endocrine therapy and discontinuation. Our results were similar with previous studies concerning the ages [35, 36]. While our findings were partly consistence with Sheppard and colleagues [29], in which the authors observed that Swedish patients younger than 40 and older than 65 are at a higher risk of treatment discontinuation. However, Hershman *et al.* reported that the adherence was rather poor among younger women [14]. Moreover, a recent published paper by Fan *et al.* also suggested that young age was identified as a risk factor for nonadherence in Taiwanese women treated with hormone therapy [37]. The poor adherence and persistence in the younger patients is mainly due to the side effects caused by endocrine therapy [38–40]. The age difference between our study and the above two studies might be explained as follows: first, we enrolled large proportions of young patients at diagnosis < 50 years (45.5% of all patients); second, Northeast of China is relatively underdeveloped compared to Western countries or even Taiwan. Lack of medical and public health infrastructure in these areas is still a challenge for communities, especially for the elderly; third, elderly women more often display less fear of cancer recurrence and health worry [41], especially in the undeveloped areas; and fourth, elderly patients are at greater risk of developing medical comorbidities (e.g. hypertension, heart disease, and/or diabetes) and cognitive impairment, therefore elderly individual are using many drugs at the same time [42]. Increasing forgetfulness, confusion, lack of understanding of treatment, and suspicions of drugs can all contribute to the failure of maintaining drug regimens [43].

Moreover, we observed that residence place influenced the endocrine treatment patterns. Shenyang is the largest city in Northeast China, however, a total of 398 patients were from suburban and rural. Patients in the suburban and rural areas may have less opportunity acquiring higher education level and are inconvenient and inadequate to visit hospitals and/or to obtain medication due to the financial problems. As mentioned in our findings, we found that patient-related factors (such as the negative attitudes, perceptions, expectations and beliefs) appeared to be critical for not initiation of endocrine treatment or discontinuation, which were in line with previous studies [44–46]. In addition, we also demonstrated that therapy-related factors (such as side effects) contributed to the non-endocrine treatment and/or discontinuation of endocrine therapy. Compared to AI only or sequentially combined with tamoxifen, tamoxifen only was a risk factor for discontinuation in the present study. Although tamoxifen is well tolerated in patients, significant and different side effects may occur during the long-term therapy, leading to the poor adherence and persistence [47, 48]. A growing number of studies have pointed out that the side effects of tamoxifen are the main cause of the low adherence [47, 49, 50]. AI has been identified to be superior to tamoxifen as hormonal therapy for postmenopausal ER+ breast cancer [51]. More interestingly, the results in the present study showed that the persistence was also lower in patients with locally advanced stage and patients without radiotherapy and/or chemotherapy. For patients with locally advanced stage, patients easily lost confidence due to the lack of education and the perception that endocrine therapy can bring much benefit. Endocrine therapy is normally performed post-chemotherapy or post-radiotherapy. For patients who received no radiotherapy and/or chemotherapy or who cannot tolerate the treatments due to the side effects, the patient's general condition were very poor and they seemed not to take a long time of endocrine therapy.

A main limitation of our study was the self-reported information from the patients. Northeast China is a relatively backward region, and we were still unable to get medication information through the medical system network. The medication information was only obtained by telephone follow-up, outpatient follow-up, and medical education conference. Although the limitation, our results indicate that the persistence of adjuvant endocrine therapy is low in women breast cancer patients in Northeast China. Non-endocrine therapy and early discontinuation serves as independent predictors for DFS and OS. Elderly women, those who live in suburban and rural areas, patients with locally advanced stage, and patients without radiotherapy and chemotherapy treatment are at high risk of non-endocrine treatment and discontinuation of endocrine treatment.

## MATERIALS AND METHODS

### Subjects and information collection

We enrolled a cohort of women who were diagnosed with breast cancer between January 2007 and December 2010 at the First Hospital of China Medical University (Shenyang, China). Demographic and clinic pathological parameters including age at diagnosis, family history of breast/ovarian cancer, history of gynecological diseases, tumor stage, lymph node metastasis involvement, types of surgery, and therapeutic modality were retrieved from patient records. Tumor stage was used to evaluate the tumor size (T1: ≤ 2 cm, T2: > 2 cm but ≤ 5 cm, and T3: > 5 cm). Types of surgery included tumorectomy, breast-conserving, and modified radical mastectomy/mastectomy plus sentinel lymph node biopsy. Endocrine therapy consisted of tamoxifen only, aromatase inhibitor (AI) only, or both. This study was approved by the Ethics Committee of our hospital, and written informed consent was obtained from each participant.

### Follow-up and definitions

Telephone follow-up was conducted at 6-month intervals for 5 years by experienced interviewers. The information included anticancer treatment after discharge and survival status. The reasons for non-endocrine treatment or early discontinuation were collected. Information on disease progression was extracted from outpatient follow-up records. The follow-up deadline was August 2016. Median follow-up time was 61 months, and the mean number of follow-up for each patient is 3.4 times. To avoid the potential the rate of lost to follow-up, a continuing medical education conference was held every six month in our hospital. Herein, continuation is defined as receiving endocrine treatment for 5 years. Early discontinuation is defined as cessation of tamoxifen and/or AI treatment within 5 years. DFS is defined as the time between diagnosis of disease and recurrence or distant metastasis. DFS was identified by clinical evidence of disease and the pathological examination (eg, punch biopsy). OS is defined as the time from diagnosis of disease to death.

### Statistical analysis

Kaplan-Meier curves were used to quantify the values of DFS and OS over time and compared using the log-rank test. Univariate and multivariate Cox proportional hazards regression models were performed to identify prognostic factors for DFS and OS of patients. Multiple logistic regression analysis was conducted to determine the possible risk factors for non-endocrine treatment and treatment discontinuation. All analyses were conducted using SPSS version 18 (SPSS Inc., Chicago, IL). A value of *P* < 0.05 was considered as significant difference.

## Abbreviations

ER+: Estrogen receptor positive
ATLAS: Adjuvant Tamoxifen: Longer Against Shorter
aTTom: trial and the Adjuvant Tamoxifen—To Offer More?
AI: aromatase inhibitor
DFS: disease-free survival
OS: overall survival
GnRHa: gonadotropin-releasing hormone agonists
SERMs: selective estrogen receptor modulators
SERDs: selective estrogen receptor own-regulators
HR: hazard rate
CI: confidence interval
MRD: modified radical mastectomy
SLN: sentinel lymph node biopsy
